# Metabolic acclimation supports higher aluminium-induced secretion of citrate and malate in an aluminium-tolerant hybrid clone of *Eucalyptus*

**DOI:** 10.1186/s12870-020-02788-4

**Published:** 2021-01-06

**Authors:** Wannian Li, Patrick M. Finnegan, Qin Dai, Dongqiang Guo, Mei Yang

**Affiliations:** 1 Guangxi Key Laboratory of Forest Ecology and Conservation, College of Forestry, Guangxi University, 100 East University Road, Nanning, 530004 Guangxi People’s Republic of China; 2 School of Biological Sciences, University of Western Australia, Perth, 6009 Australia; 3 Guangxi Forestry Rearch Institute, Nanning, 530002 Guangxi People’s Republic of China

**Keywords:** *Eucalyptus*, Aluminum tolerance, Citrate, Malate, Metabolizing enzymes

## Abstract

**Background:**

*Eucalyptus* is the main plantation wood species, mostly grown in aluminized acid soils. To understand the response of *Eucalyptus* clones to aluminum (Al) toxicity, the Al-tolerant *Eucalyptus grandis × E. urophylla* clone GL-9 (designated “G9”) and the Al-sensitive *E. urophylla* clone GL-4 (designated “W4”) were employed to investigate the production and secretion of citrate and malate by roots.

**Results:**

*Eucalyptus* seedlings in hydroponics were exposed to the presence or absence of 4.4 mM Al at pH 4.0 for 24 h. The protein synthesis inhibitor cycloheximide (CHM) and anion channel blocker phenylglyoxal (PG) were applied to explore possible pathways involved in organic acid secretion. The secretion of malate and citrate was earlier and greater in G9 than in W4, corresponding to less Al accumulation in G9. The concentration of Al in G9 roots peaked after 1 h and decreased afterwards, corresponding with a rapid induction of malate secretion. A time-lag of about 6 h in citrate efflux in G9 was followed by robust secretion to support continuous Al-detoxification. Malate secretion alone may alleviate Al toxicity because the peaks of Al accumulation and malate secretion were simultaneous in W4, which did not secrete appreciable citrate. Enhanced activities of citrate synthase (CS) and phosphoenolpyruvate carboxylase (PEPC), and reduced activities of isocitrate dehydrogenase (IDH), aconitase (ACO) and malic enzyme (ME) were closely associated with the greater secretion of citrate in G9. PG effectively inhibited citrate and malate secretion in both *Eucalyptus* clones. CHM also inhibited malate and citrate secretion in G9, and citrate secretion in W4, but notably did not affect malate secretion in W4.

**Conclusions:**

G9 immediately secrete malate from roots, which had an initial effect on Al-detoxification, followed by time-delayed citrate secretion*.* Pre-existing anion channel protein first contributed to malate secretion, while synthesis of carrier protein appeared to be needed for citrate excretion. The changes of organic acid concentrations in response to Al can be achieved by enhanced CS and PEPC activities, but was supported by changes in the activities of other enzymes involved in organic acid metabolism. The above information may help to further explore genes related to Al-tolerance in *Eucalyptus*.

## Background

Species of *Eucalyptus* are typical fast-growing hardwood trees. Their wide planting has had an important impact on the world timber market due to the high yield of lumber. *Eucalyptus* is naturally adapted to various environmental conditions, including acid soils [1, 2]. Thus, *Eucalyptus* plantations have been established in acidic soils that are widely distributed in tropical and subtropical climate zones, including in south China. However, it is widely recognized that Al^3+^ is solubilized into the soil solution and is rhizotoxic to plants when soil pH is below 5.0 [3]. Thus, it is of great interest that acid soils caused no reduction in *Eucalyptus* productivity [2].

Various strategies have evolved in higher plants to alleviate Al phytotoxicity, ranging from external repulsion mechanisms to **i**nternal endurance mechanisms [4–6]. Aluminium-induced production of organic acids is considered to be one of the key tolerance mechanisms for detoxifying both internal and external Al [7, 8]. The accumulation of Al in root tips usually leads to rapid inhibition of root growth within minutes to hours by affecting the absorption of nutrients and disrupting other physiological processes [9]. Several studies have suggested that species of *Eucalyptus* have higher tolerance to Al toxicity than other tree species such as *Quercus robur*, *Pinus radiata* and *Melaleuca cajuputi*, and may even benefit from low concentrations of Al [10–12]. Moreover, species of *Eucalyptus* and their clones vary in their Al tolerance and response to Al in acidic environments [12–17]. As in other plants, the exudation of low molecular weight organic acids from the roots of several species or genotypes of *Eucalyptus* may be an important determinant for Al tolerance and may allow *Eucalyptus* to grow and yield well in acidic soils [13, 18, 19]. In recent years, asexual hybrid clones of fast-growing *Eucalyptus* have been the main source of high-yielding plantation stock. Knowledge of the role of organic acids in Al-tolerance of these superior hybrid clones grown in aluminized acidic soils is limited and needs further elucidation.

Citrate and malate are the main Al-chelating organic acids that confer tolerance to Al in plants. The types of organic acids and their transportation pathways that are induced by Al vary among different plant species and genotypes [20]. Aluminium-activated organic acid secretion is driven by passive outward movement down a concentration gradient that requires anion channels. In some crops, the activity of organic acid anion channels were more rapidly induced in Al-tolerant genotypes than in sensitive genotypes [21]. This rapid induction of organic acid secretion is mainly due to pre-existing membrane-localized anion channel proteins encoded by *ALMT* (Al-activated malate transporter) or *MATE* (Al-activated citrate transporter) genes [22, 23]. When there is a time delay in Al-induced organic acid secretion, new proteins involved in organic acid secretion can be synthesized within hours [2, 24]. Sawaki et al. [1] showed that in *Eucalyptus camaldulensis* citrate excretion through citrate-transporting EcMATE proteins was an important Al-tolerance mechanism. However, more work is needed on Al-induced secretion of organic acids from the root to determine whether there are other key components that may impact on the secretion process.

Low molecular weight organic acids involved in tricarboxylic acid (TCA) cycle, such as citrate and malate, are mainly synthesized in mitochondria. The synthesis and secretion of Al^3+^-induced organic acids are affected by changing activities of enzymes involved in Organic Anion (OA) metabolism and altering expression of corresponding genes [25], but these effects vary depending on plant species and genotype. In soybean, a cytosolic malic enzyme (ME) encoded by *GmME1* contributed to increased internal malate and citrate concentrations and their efflux, conferring higher Al resistance [26]. In alfalfa, overexpression of genes encoding citrate synthase (CS) and malate dehydrogenase (MDH) led to increased concentrations and exudation of citrate and malate, and increased Al resistance [27]. Thus, increasing enzyme activities related to increasing malate and citrate production are effective in conferring Al tolerance to plants.

In previous research, we determined that *Eucalyptus* hybrid *E. grandis* × *E. urophylla* GL-9 (designated G9 here) was more Al-tolerant than *E. urophylla* GL-4 (designated W4 here) [28–30]. Lima et al. [16] reported that *E. grandis* possessed higher Al-tolerance than *E. platyphylla* due to a minor increase in reactive oxygen species and fewer alterations to stress indicators when exposed to high Al. We concluded that *E. grandis* × *E. urophylla* was more Al-tolerant than *E. urophylla* based on the low level induction of physiological antioxidant indicators [29]. Based on these results, we inferred that hybridization contributed to Al-tolerance in *E. grandis* × *E. urophylla*, and provided it with the ability to acclimate to the acidic soils found throughout south China. Based on previous identification of organic acids secreted by *Eucalyptus* roots [2, 13, 18, 19], we hypothesized that citrate would be the main organic acid involved in Al detoxification in G9, and that malate may not have an important role. In addition to expecting higher CS activity in Al-tolerant G9 than W4, we also hypothesized that changes in other enzymatic activities associated with OA metabolism would support an increase in citrate accumulation. Such metabolic acclimations would provide further information to support strategies to increase Al tolerance in *Eucalyptu*s and would be expected to be applicable to other species.

## Results

### Accumulation of Al in roots of *Eucalyptus*

**Fig. 1:**
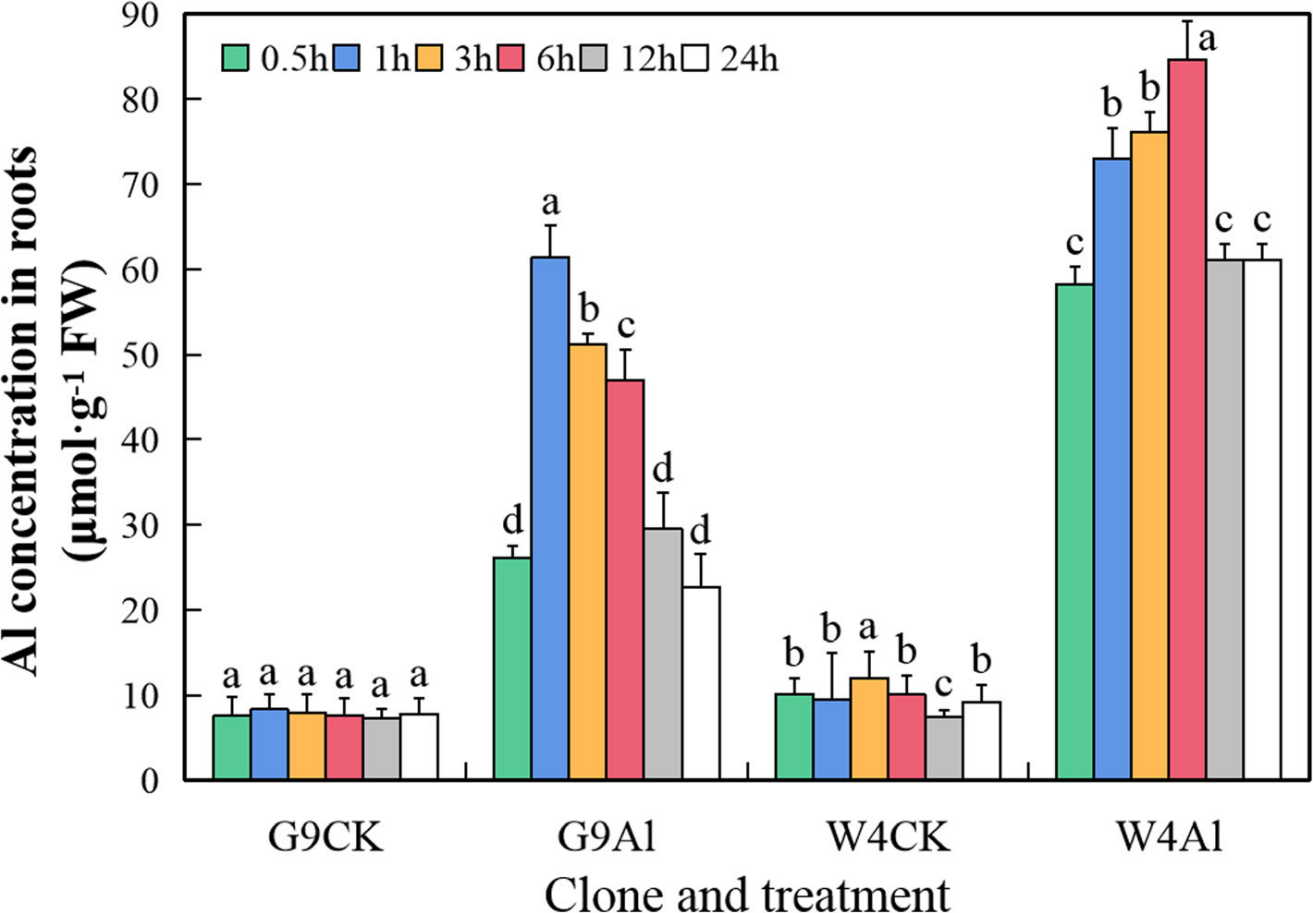
Aluminum concentration in root tips of *E. grandis* × *E. urophylla* G9 and *E. urophylla* W4 at the indicated times after addition of Al. Bars represent means ± standard errors (*n* = 3). Different letters above the bars indicate a significant difference at *P* < 0.05. CK, non-Al-treated control; Al, aluminium treated

Treatment with Al over a 24 h time course caused significantly higher Al concentrations in root tips of both G9 and W4 genotypes compared to the absence of Al (Fig. 1). The Al concentration peaked at 1 h in G9 root tips, and then declined by about 65% over the remainder of the time course. In W4 root tips, the Al concentration rose more rapidly than in G9 and continued to increase until around 6 h, after which it declined somewhat. The Al concentration was higher in W4 root tips than in G9 root tips at all points of the time course. The lower accumulation of Al in G9 root tips indicated that the degree of Al exclusion differed between the two *Eucalyptus* clones that had differential tolerance to Al. This result was similar to that in our previous study on Al accumulation in 7-month-old soil-grown *Eucalyptus* seedlings exposed to Al for 4 months. The Al concentration in roots, stems and leaves of G9 was lower than those of W4 [31]. Taken together, these results suggested that G9 may have a greater ability to exclude Al from the interior of the root by chelating Al around its rhizosphere, thereby reducing Al absorption.

### Al-induced secretion of organic acids from roots of *Eucalyptus*

**Fig. 2:**
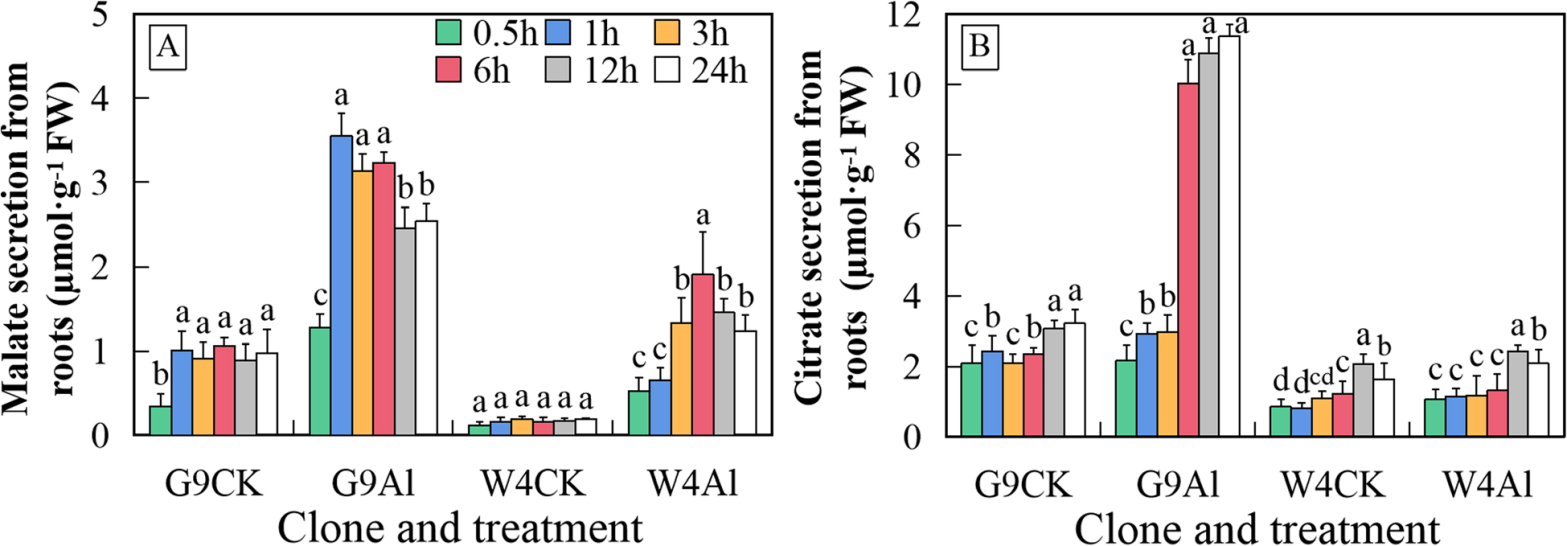
Aluminium-induced malate (**a**) and citrate (**b**) excretion from roots of *E. grandis* × *E. urophylla* G9 and *E. urophylla* W4 into growth media at the indicated times after initiation of Al treatment. Growth media containing root exudates was sampled at each time point after the addition of Al at the same time that the seedlings were harvested. Data are means ± standard error (*n* = 3). Different letters above the bars indicate a significant difference at *P* < 0.05. CK, non-Al-treated control; Al, aluminium treated

The Al-inducible malate and citrate secretion from roots was compared between the contrasting clones over a 24 h time course (Fig. 2). The amount of malate secreted from roots was generally lower for each clone than the amount of citrate secreted in both the non-Al-treated controls (CK) and the Al-treated plants. Clone G9 had a higher level of organic acid secretion than W4 at all time points after the start of the Al exposure. The accumulation of malate in G9 peaked around 1 h after exposure to Al and then decreased gradually, but was still more than 2-fold higher than the concentration in the absence of Al after 24 h. (Fig. 2a). In clone W4, by contrast, the accumulation of malate reached its maximum after 6 h of Al treatment. The maximum in W4 was only about 60% of that in G9. Citrate accumulation for G9 was activated after 3 h of Al exposure and peaked by 6 h, while there was no significant change in citrate accumulation in W4 through 24 h (Fig. 2b). These results indicated that the secretion mechanisms of these two organic acids differed between the tolerant and sensitive genotypes of *Eucalyptus*.

### Effect of inhibitors on organic acid secretion by *Eucalyptus* clones with contrasting Al tolerance

**Fig. 3:**
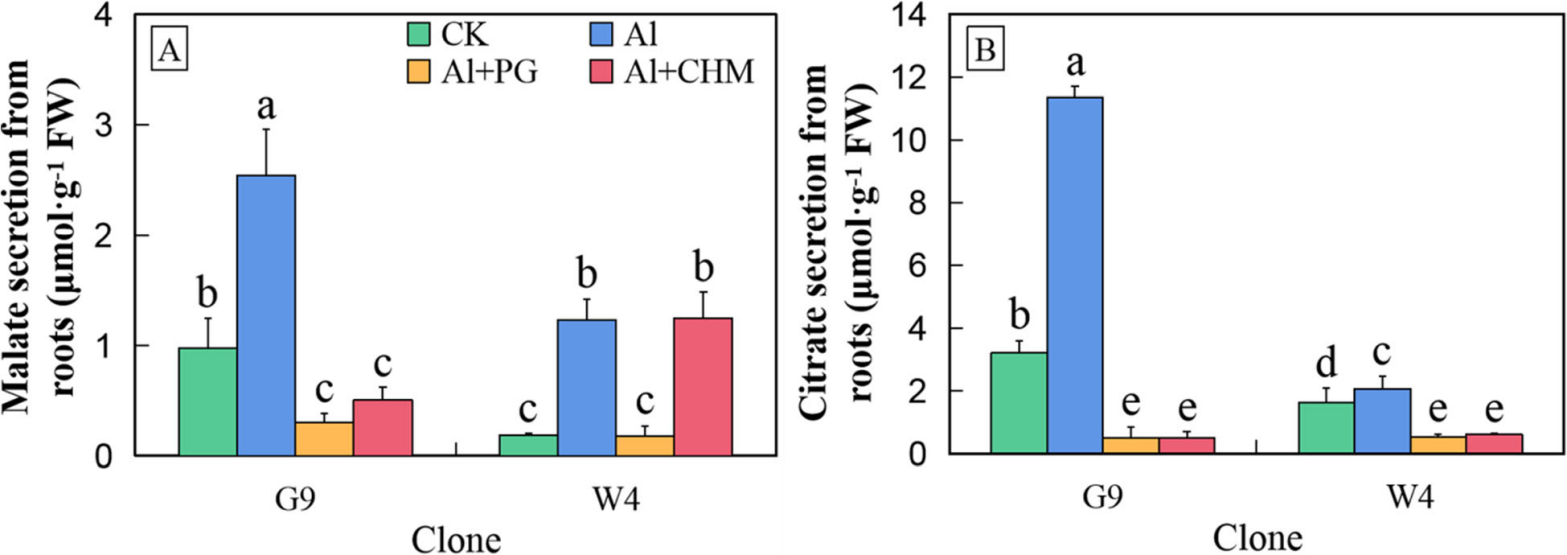
Malate (**a**) and citrate (**b**) secreted from roots of *E. grandis* × *E. urophylla* G9 and *E. urophylla* W4 after 24 h of exposure to aluminium in the absence or presence of the anion channel inhibitor phenylglyoxyl (PG) or the protein synthesis inhibitor cycloheximide (CHM). Bars represent means ± standard errors (*n* = 3). Different letters above the bars indicate a significant difference at *P* < 0.05. CK, non-Al-treated control

To investigate the secretory pathways for citrate and malate from roots after Al treatment, the anion-channel inhibitor phenyglyoxal (PG) and the protein-synthesis inhibitor cycloheximide (CHM) were added at the start of a 24-h Al treatment (Fig. 3). Phenyglyoxal fully inhibited Al-induced malate secretion in both G9 and W4. In fact, PG also inhibited the non-Al-inducible secretion of malate in G9 to the same low background level observed in W4. The impact of CHM treatment on malate secretion in G9 was similar to that seen for PG treatment. However, CHM had no effect on the Al-induced secretion of malate in W4. The Al-induced secretion of citrate in both G9 and W4 was inhibited by CHM to the same low level, which was below the level of citrate secretion in the absence of Al.

### Organic acid concentrations in root tips of *Eucalyptus* clones with contrasting Al tolerance

**Fig. 4:**
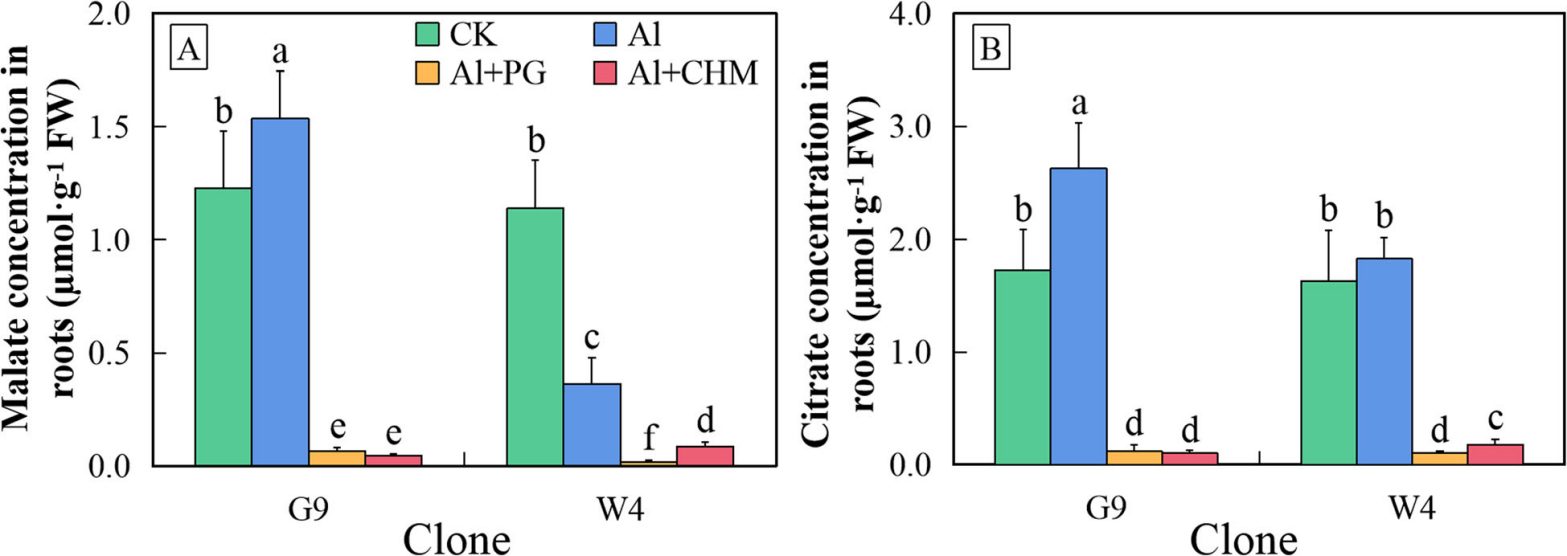
Malate (**a**) and citrate (**b**) concentrations in root tips of *E. grandis* × *E. urophylla* G9 and *E. urophylla* W4 after 24 h treatment with aluminium in the absence or presence of the anion channel inhibitor phenylglyoxyl (PG) or the protein synthesis inhibitor cycloheximide (CHM). Bars represent means ± standard errors (*n* = 3). Different letters above the bars indicate significant differences at *P* < 0.05. CK, non-Al-treated control

No significant differences were found in the internal concentrations of malate or citrate in root tips in the absence of Al (Fig. 4). After 24-h exposure to Al, the citrate and malate concentrations in G9 increased by about 50 and 25%, respectively, compared to the control. Thus, G9 root tips acclimated to Al stress by accumulating citrate and malate, which then allowed their secretion to the outside of the root. Meanwhile, the concentration of malate in W4 root tips upon exposure to Al decreased by about 70%, but no change was observed for citrate. The presence of PG or CHM significantly inhibited the accumulation of both malate and citrate in the root tips of both clones to below the level observed in the absence of Al. The inhibition in Al-induced accumulation of the organic acids was over 90% in all cases except for the inhibition of malate accumulation in W4, which was 76% from a lower starting level.

### Activities of acid-metabolizing enzymes in root tips of *Eucalyptus* clones with contrasting Al tolerance

**Fig. 5:**
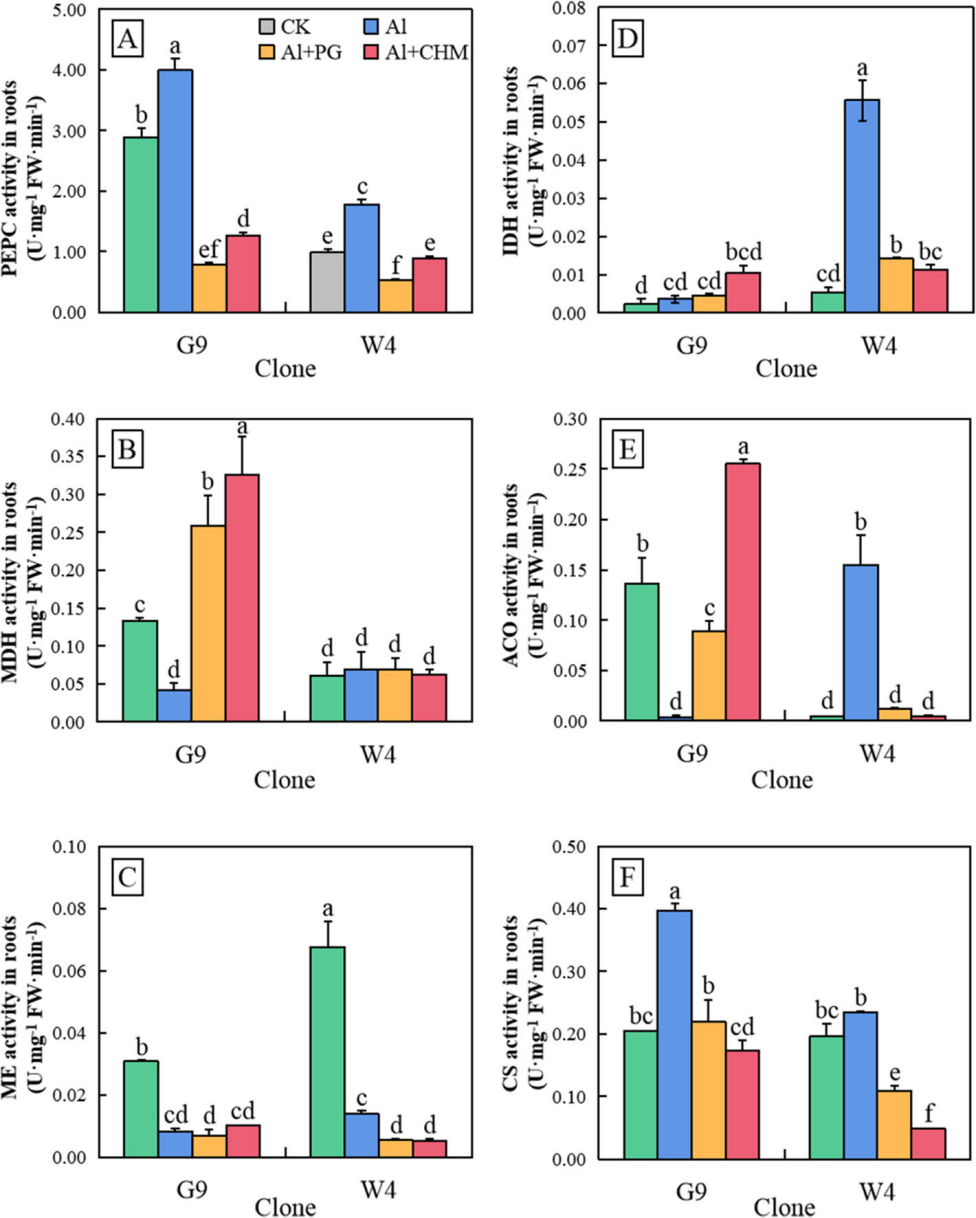
Activities of PEPC (**a**), MDH (**b**), ME (**c**), IDH (**d**), ACO (**e**) and CS (**f**) in root tips of *E. grandis* × *E. urophylla* G9 and *E. urophylla* W4 after 24 h treatment with aluminium in the absence or presence of the anion-channel inhibitor phenylglyoxyl (PG) or the protein-synthesis inhibitor cycloheximide (CHM). Bars represent means ± standard errors (*n* = 3). Different letters above the bars indicate significant differences at *P* < 0.05. CK, non-Al-treated control

The activities of phosphoenolpyruvate carboxylase (PEPC), MDH, ME, isocitrate dehydrogenase (IDH), aconitase (ACO) and CS were examined in root tips of plants exposed to Al and inhibitors (Fig. 5). PEPC, MDH and ME are important enzymes associated with the metabolism of malate. In the absence of Al, the activity of PEPC was higher in root tips of G9 than of W4. There was a significant increase in PEPC activity in root tips of both clones after Al treatment, but the activity was below non-treated control levels when either PG or CHM were included in the Al treatment. In G9, the addition of inhibitors resulted in 80% lower PEPC activity compared to the addition of Al alone. MDH and ME activities were significantly lower in G9 after Al treatment than in the absence of Al treatment, as was the activity of ME in W4. In contrast, the activity of MDH in W4, which was already as low as in Al-treated G9, was unaffected by Al treatment. Interestingly, compared with the treatment with Al alone, the activity of MDH in G9 increased significantly after the addition of both PG and CHM, while the activity of MDH in W4 and ME in both clones remained unchanged by the addition of these inhibitors.

IDH, ACO and CS are closely associated with citric acid metabolism. Compared to the non-Al treatment, IDH and ACO activities in W4 root tips exposed to Al were significantly higher, while CS activity was unchanged. In contrast, in root tips of Al-treated G9, IDH activity was unchanged, ACO activity was much lower and CS activity was 2-fold higher than in the absence of Al treatment. The increased activity of CS may be one of the main reasons for the higher Al-induced accumulation and secretion of citrate in G9. Both PG and CHM had no impact on the low activity of IDH in root tips of Al-treated G9, but repressed the Al-induced increase in CS activity and relieved the Al-induced loss of ACO activity. In Al-treated W4 root tips, the two inhibitors abolished the Al-induced increase in IDH and ACO, and lowered the CS activity to below that in the untreated control.

## Discussion

### An Al-tolerant *Eucalyptus* hybrid clone has enhanced accumulation and exudation of malate and citrate

The available evidence indicates that the Al-induced secretion of organic acids from roots may lead to the detoxification of Al in higher plants [32, 33]. A role for organic acids leading to Al tolerance in *Eucalyptus* has been observed previously [12, 18, 19]. The lower root tip concentration of Al coupled with the higher root secretion of citrate and malate in Al-tolerant *E. grandis* × *E. urophylla* clone G9 than that in Al-sensitive *E. urophylla* clone W4 suggested that secretion of these two organic acids was involved in the increased tolerance to Al in G9. This trait was consistent with the results reported for Al-tolerant *E. camaldulensis* [12, 18, 34]. Tahara et al. [12] documented in *E. camaldulensis* that citrate had the strongest capacity to bind Al among citrate, oxalate, malate and phosphate. Thus, it was likely that the Al-stimulated accumulation and secretion of citrate was the main underlying mechanism contributing to detoxification of Al by *Eucalyptus* roots, particularly in Al-tolerant genotypes. However, Silva et al. [19] put forward the hypothesis that Al tolerance was due to the internal detoxification of Al by complexation with malate. These conflicting conclusions left the role of malate in *Eucalyptus* tolerance unclear. Adding to the complexity, the types of organic acids produced and released in response to Al may vary among *Eucalyptus* species [34], as do the quantities, as shown here for malate and citrate. The features of malate and citrate accumulation and secretion in Al-tolerant hybrid clone G9 and Al-sensitive parental clone W4 have provided further clues for the identification Al-induced genes or proteins.

### Newly synthesized carrier proteins involved in citrate secretion, but malate secretion facilitated by a pre-existing anion channel in *E. grandis* × *E. urophylla*

A rapid release of organic acid in response to exposure to Al would suggest that pre-existing anion transporters on the plasma membrane quickly initiated organic acid secretion without the need to produce new proteins; however, a lag in the release of organic acids could indicate that gene expression and/or protein synthesis was required [33, 35, 36]. There was no significant delay in malate secretion by G9, followed by an increase in the secretion of citrate after a lag period of more than 3 h. In contrast, in W4, there was a lag of more than an hour after Al exposure before malate secretion became apparent, while Al exposure did not induce the production or secretion of citrate. Thus, 24 h after exposure to Al, the synthesis and secretion of malate and citrate by G9 was much greater than in W4.

Both PG and CHM significantly reduced the Al-induced secretion and internal concentration of citrate in roots of both *Eucalyptus* clones as well as the malate concentration in G9. However, CHM had no impact on malate secretion in W4, indicating that there are different pathways operating for citrate and malate secretion in response to Al in the two clones. Generally, Al-tolerant species or genotypes had stronger induction and higher quantities of carrier proteins on membranes inside root cells and anion channel proteins on the plasma membrane of root cells, than Al-sensitive genotypes [37, 38]. If organic acid synthesis and transport require the involvement of newly synthesized carrier proteins, an obvious lag of several hours before secretion would be apparent, while pre-existing anion channel proteins would allow organic acids to be secreted out of the root more quickly [39, 40]. Therefore, in G9, it seems likely that pre-existing anion channel proteins facilitated the immediate secretion of malate, while a new carrier protein apparently had to be produced before citrate could be transported out of the roots. Anion channel proteins, such as ALMT and MATE/AACT, are localized to the plasma membrane of root cells and transport their substrates to rapidly facilitate organic acid release at phytotoxic concentrations of Al^3+^ [41, 42]. Furthermore, numerous genes encoding OA transporters have been found to increase OA secretion and to be involved in Al detoxification [43, 44]. Sawaki et al. [1] reported that Al-induced excretion of citrate by *E. camaldulensis* roots was associated with higher expression of EcMATE on the plasma membrane, and that the ectopic expression of EcMATE in tobacco hairy roots enhanced Al-responsive citrate excretion, providing further insight into the molecular mechanism underlying Al resistance in *Eucalyptus* and the potential for genetic improvement of *Eucalyptus*. However, other components remain to be revealed, particularly the new protein-coding genes and their functions in organic acid synthesis and transport. For instance, the delay in citrate secretion found in G9 was likely due to the need to produce new proteins involved in the synthesis and delivery of citric acid. For W4, there was no change in citrate secretion in response to exposure to Al, while malate secretion was delayed and did not reach its maximum level for 6 h. Moreover, CHM had no effect on the secretion of malate, but did inhibit its accumulation, indicating that W4 does not lack the capacity to release malate, but rather was restricted in its ability to produce malate.

Increasing organic acids exudation may not be the only effective way to enhance Al resistance of *Eucalyptus*. We speculate that other organic substances might be involved in detoxifying Al in some *Eucalyptus* genotypes. A consequence of Al tolerance in *Eucalyptus* was the maintenance of nutrients and photosynthesis [17, 45]. A new low-molecular-weight Al-binding ligand from roots, oenothein b, contributed to Al tolerance in *E. camaldulensis* [12, 46]. In addition, a number of allelochemicals were detected in *E. grandis* roots and soil by GC-MS [47, 48]. Many of these chemicals are involved in either primary or secondary plant metabolism and plant defense processes [49]. These process may interfere with the secretion of low molecular organic acids, or their products may form complexes with Al. For example, one study found that phenolic compounds could be involved in Al detoxification forming strong complexes with Al ions in the cytoplasm of woody plants including *E. viminalis* Labill [50]. In addition, transcriptome analysis has revealed that genes associated with flavonoid and phenylpropanoid biosynthetic pathways have key roles in the response of roots of *Cunninghamia lanceolata* (lamb.) hook. to Al [51]. All these findings encourage further research to identify compounds and the related genes that confer Al tolerance to hybrid clones of *Eucalyptus*, including the contributions made by allelopathic compounds and other root exudates.

### Secretion and accumulation of citrate and malate in hybrid clone *E. grandis* × E. urophylla GL-9 were closely linked with changes in CS and PEPC activities

We observed that CS and PEPC activities in root tips of both clones were markedly induced by Al, while ME activity was significantly decreased. Together, these changes likely contribute to the increased biosynthesis of organic acids by feeding carbon skeletons into the TCA cycle [52]. The balance between synthesis or catabolism of Al-induced citrate and malate was regulated by shifts in activities of various metabolic enzymes that together contributed to accumulation of these organic acids to increase Al tolerance in *Eucalyptus*. Additionally, the addition of inhibitors (PG and CHM) directly or indirectly caused changes in enzyme activities involved in organic acid metabolism.

In the case of *E. urophylla* clone W4, decreased ME activity may play a greater role in the lower accumulation of malate upon exposure to Al, since MDH activity was unchanged. Meanwhile, the activities of ACO and IDH were significantly increased by Al exposure, which may underlie the lack of an increase in citrate. In *E. grandis × E. urophylla* clone G9, increased synthesis and secretion of malate seemed to be supported by decreased ME and MDH activity to prevent malate metabolism. We speculate that genes encoding ME may contribute to increased internal malate and citrate concentrations, leading to exudation of these organic acids to confer higher Al resistance, as in soybean [26]. CS is typically regarded as the main enzyme necessary to increase synthesis and secretion of citrate in roots of Al-tolerant plants, such as rye [53], *Paraserianthes facataria* [54], and soybean [55]. Moreover, transcript levels specifying CS, ALMT and MATE in the root apex of an Al-tolerant cultivar of alfalfa were higher than in an Al-sensitive cultivar [27]. However, Ikka et al. [34] found that the Al-induced increase in citrate concentration in roots of *E. camaldulensis* was not due to increased CS activity, but was dependent on reduced ACO activity, which would suppress citrate catabolism*.* Recently, Teng et al. [56] reported that CS, PEPC and IDH may play important roles in organic acid biosynthesis and degradation in *Eucalyptus.* Our study indicated that the increased synthesis and secretion of citrate that contributed to increase Al-tolerance in *E. grandis × E. urophylla* was likely achieved by increasing the activities of PEPC and CS, and decreasing the activity of ACO. These three enzymes may be involved in creating the balance between the secretion of malate and citrate in the roots of plants exposed to Al.

From the above, it is clear that key enzymes regulating OA synthesis and exudation vary among of *Eucalyptus* genotypes. Alterations in the expression of the corresponding genes can affect OA synthesis and exudation resulting in changes in Al tolerance [57]. Some effort has been made in plants to increase the expression of enzymes such as PEPC, CS and MDH by introducing genes encoding these enzymes, for example, in tobacco, alfalfa and canola. Overexpression of these genes would be expected to increase organic acid metabolism and may produce a new citrate synthesis pathway that would contribute to increased Al tolerance in transgenic plants [58–60]. For example, in transgenic canola, overexpression of a CS gene not only led to increased citrate synthesis and exudation, but also changed malate metabolism, which may improve tolerance to Al toxicity [57, 61, 62]. Since previous studies have indicated that the synthesis of organic acids could be increased by regulating the expression of genes encoding enzymes involved in OA synthesis or transporters involved in OA secretion, transgenic approaches can be expected to provide higher Al tolerance in plants, including *Eucalyptus*.

## Conclusion

It appears that both citrate and malate contributed to Al tolerance in *Eucalyptus* and that both accumulation and secretion of these organic acids were involved in Al detoxification. The superior performance of hybrid clone *E. grandis* × *E. urophylla* for high yield on aluminized acidic soils was closely associated with increased capacity to release both citrate and malate. Citrate had a more important role in the response to Al in *E. grandis* × *E. urophylla* than in *E. urophylla*. In addition, PG and CHM treatments indicated that both anion channel proteins and increased carrier protein synthesis were involved in Al-induced secretion of citrate in Al-tolerant *E. grandis* × *E. urophylla*, but the secretory pathway for malate remained unclear. The enhanced activities of CS and PEPC and the reduced activities of IDH, ACO and ME contributed to Al-induced accumulation and secretion of citrate and malate, demonstrating that metabolic adaptations are associated with Al-tolerance in *E. grandis* × *E. urophylla*. More effort is needed to identify and isolate the genes associated with organic acid transport in *Eucalyptus*, or introduce exogenous genes to make specific enzymes overexpressed and have high activity, further advance Al-tolerance in superior hybrids of *Eucalyptus*. Meanwhile, other compounds which could be involved in Al detoxification also need further investigation and elucidation of their regulatory mechanism.

## Methods

### Plant material

Two clones of *Eucalyptus*, Al-tolerant *E. grandis* × *E. urophylla* GL-9 (Voucher number: 桂S-SC-EGU-023-2011; designated G9) and Al-sensitive *E. urophylla* GL-4 (Voucher number: 桂S-SC-EU-022-2011; designated W4) were used in this study. Two-month-old seedlings from culture were provided by the Guangxi Forestry Research Institute, Nanning, China. Guo Dongqiang, a senior engineer and researcher formally identified the two clones [see Additional files 1, 2 and 3]. Seedlings of similar appearance and size were placed in 2 L plastic buckets containing nutrient solution with 10 seedlings per bucket. All solutions were prepared with deionized water. For acclimating plants to the hydroponic culture system before Al treatment, seedlings were pre-cultured in 20% nutrient solution, pH 5.0 for 3 days, then in 50% nutrient solution, pH 4.5 for a further 3 days. Acclimated seedlings were transferred to 100% nutrient solution, pH 4.0 for 7 days. The composition of 100% nutrient solution was 6 mM KNO_3_, 5 mM Ca (NO_3_)_2_, 1 mM MgSO_4_, 2 mM NH_4_H_2_PO_4_, 20 μM Fe-EDTA, 31.25 μM H_3_BO_3_, 2 μM MnCl_2_, 2 μM ZnSO_4_, 0.5 μM CuSO_4_ and 0.065 μM (NH_4_)_6_Mo_7_O_24_. The pH was adjusted with 2 mM HCl. All nutrient solutions were renewed every 2 days. Prior to solution replacement, seedlings were sterilized with 0.1% (v/v) carbendazim for 20 min to inhibit microorganisms. Air pumps were used to continuously aerate the seedlings in hydroponics at 50 L air h^− 1^.

### Aluminum and inhibitor treatments

After 7 days of culture in complete nutrient solution, pH 4.0, seedlings were transferred to 2 L fresh nutrient solution, pH 4.0, supplemented with or lacking 4.44 mM Al^3+^ from AlCl_3_•6H_2_O with ten seedlings per pot. Each treatment was carried out in triplicate (3 × 10 plants). The growth solution and roots were harvested from three pots at 0.5 h, 1 h, 3 h, 6 h, 12 h, and 24 h from the start of exposure to Al^3+^. The internal concentration of citrate and malate and their concentration in the growth medium were determined at these time points. The activities of organic acid-metabolizing enzymes inside root tips were determined after 24 h of Al treatment.

To identify potential factors involved in organic acid secretion, the impact of the protein synthesis inhibitor CHM, and the anion channel blocker PG were determined by cultivating the seedlings as above in solutions supplemented with 0.5 mg L^− 1^ CHM and 0.5 mg L^− 1^ PG with 0 or 4.44 mM Al^3+^ for 24 h. The secretion of citrate and malate from the roots was determined after 24 h.

### Determination of organic acids in root exudates

Samples were prepared following the methods of Wang et al. [63], with some modifications. The collected hydroponics solution was filtered through a mixed fiber membrane to obtain 50 mL filtrate. The filtrate was passed through a cation exchange column (15 mm × 11 cm, 5 g Amerlite IR-120 resin), followed by an anion exchange column (2 g Dowex 1-X8 resin). The organic acids bound to the anion exchange column were eluted with 2 M HCl. The eluent was condensed to dryness at 40 °C by rotary evaporation (R215, Buchi, Switzerland). The dried eluent was re-dissolved into 1 mL of Milli-Q water, and filtered (0.45 μm membrane filter). The filtered solution was analyzed for citrate and malate using ion chromatography (ICS-5000 Ion Chromatography system with 4 × 250 mm AS11-HC analytical column and 4× 50 mm AS11-HC guard column, Dionex, USA).

### Analysis of malate and citrate in root tips

Harvested roots were rinsed with Milli-Q water to remove the hydroponics solution. The distal 2 cm containing the root apices were excised to extract and assay the internal concentration of citrate and malate following the methods of Dong et al. [64] and Tahara et al. [12] with some modifications. A total weight of 0.2 g for each sample was ground under liquid nitrogen before adding 1.5 mL ice-cold 4% (v/v) HClO_4_ into the powder and gently homogenizing. The mixture was thawed slowly on ice into a suspension and allowed to stand for 30 min, followed by centrifugation at 20,000×g at 4 °C for 10 min. The supernatant was passed through an ion exchange column (l5mm × 11 cm) filled with cation exchange resin (Amerlite IR-120 resin, H^+^ form, USA) to remove cations, and was then passed through the a pretreatment column (RP18 column, Dionex, USA) to absorb plant pigments. The extracted malate and citrate in roots were determined by ICS as described above.

### Determination of Al in root tips

Roots were rinsed with Milli-Q water three times. The 3 cm at root apices were excised and dried at 80 °C before grinding to a fine powder. Powdered root tips (100 mg) were digested in 10 mL HNO_3_: HClO_4_ (5: 1 v/v) until the solution was clear. The digest was diluted to 25 mL with Milli-Q water and the Al concentration in the solution was immediately determined by inductively coupled plasma atomic emission spectroscopy (5100 ICP- OES, Agilent Technologies, USA).

### Activity measurement of organic acid-metabolizing enzymes

After plants were cultured for 24 h in the presence or absence of Al, the activities of PEPC, MDH, CS, NADP-dependent IDH, NADP-dependent NADP-ME and ACO were measured. Based on the methods of Chen et al. [65] and Yu et al. [38], 200 mg of fresh root apices were homogenized in ice-cold extraction buffer containing 50 mM HEPES-NaOH, pH 7.5, 5 mM MgCl_2_, 5 mM EDTA, 10% (v/v) glycerol, 0.1% (v/v) Triton X-100, 1% (w/v) PVPP (cross-linked polyvinylpyrrolidone) and 5 mM dithiothreitol. After clarification by centrifugation at 15,000×g for 15 min at 4 °C, the supernatant was used for enzyme activity determination. The activities were measured in a 3 mL reaction mixture using spectrophotometric assays described by Jenner et al. [66], Chen et al. [65] and Ikka et al. [34]. The activities of all enzymes were determined at 340 nm, except CS activity was determined at 412 nm.

### Data analysis

Each treatment was done with three biological replicates of 10 plants each. Statistical analysis was performed using SPSS software package. All data between different treatments was compared using one-way analysis of variance with LSD test and significant differences between the means of two treatments were determined using the Duncan test at *P* ≤ 0.05.

## Supplementary Information

**Additional file 1.** Certification of superior varieties of forest tree (*Eucalyptus grandis* × *Eucalyptus urophylla*).

**Additional file 2.** Certification of superior varieties of forest tree (*Eucalyptus urophylla*).

**Additional file 3.** Certification of *Eucalyptus* from Guangxi forestry research institute.

## Data Availability

The datasets used and analysed during the current study available from the corresponding author on reasonable request.

## Abbreviations

CHM: Cycloheximide
PG: Phenylglyoxal
CS: Citrate synthase
PEPC: Phosphoenolpyruvate carboxylase
IDH: NADP-dependent isocitrate dehydrogenase
ACO: Aconitase
ME: NADP-dependent malic enzyme
ALMT: Al-activated malate transporter
MATE: Al-activated citrate transporter
TCA: Tricarboxylic acid
OA: Organic anion
MDH: Malate dehydrogenase
CK: Control check
AACT: Aluminum-activated citrate transporter
EDTA: Ethylene diamine tetraacetic acid
NADP: Nicotinamide adenine dinucleotide phosphate
PVPP: Crosslinked polyvinylpyrrolidone
DTT: Dithiothreitol.
