# Supplementary material for: Metabolic acclimation supports higher aluminium-induced secretion of citrate and malate in an aluminium-tolerant hybrid clone of Eucalyptus
Source: BMC Plant Biol. 2021 Jan 6;21:14. doi: 10.1186/s12870-020-02788-4 (PMC7789223; doi:10.1186/s12870-020-02788-4)
Supplement: Supplementary file 1 — Additional file 1. Certification of superior varieties of forest tree (Eucalyptus grandis × Eucalyptus urophylla). [file 12870_2020_2788_MOESM1_ESM.pdf]

Title：Certification of superior varieties of forest tree (*Eucalyptus grandis* × *Eucalyptus urophylla*).

林木良种证

（审 定）

良种名称 桉树广林巨尾桉 9 号

树种 巨尾桉

学名 *Eucalyptus grandis×Eucalyptus urophylla*

良种编号 桂 S-SC-EGU-023-2011

适宜推广生态区域

北纬 24°以南，海拔 500m 以下的轻霜或无霜区域，酸性或微酸性土壤。

申请人 广西林业科学研究院

选育人 广西林业科学研究院

编号：（桂 SY ）第023号 发证机关 广西壮族自治区林木品种审定委员会

2011 年 12 月 31 日
