# Supplementary material for: Metabolic acclimation supports higher aluminium-induced secretion of citrate and malate in an aluminium-tolerant hybrid clone of Eucalyptus
Source: BMC Plant Biol. 2021 Jan 6;21:14. doi: 10.1186/s12870-020-02788-4 (PMC7789223; doi:10.1186/s12870-020-02788-4)
Supplement: Supplementary file 2 — Additional file 2. Certification of superior varieties of forest tree (Eucalyptus urophylla). [file 12870_2020_2788_MOESM2_ESM.pdf]

Title：Certification of superior varieties of forest tree

(*Eucalyptus urophylla*).

林木良种证

（审　定）

良种名称　桉树广林尾叶桉 4 号

树种　尾叶桉

学名　***Eucalyptus urophylla***

良种编号　桂 S-SC-EU-022-2011

适宜推广生态区域

北纬 **24 °** 以南，海拔 **500m** 以下的轻霜或无霜区域，酸性或微酸性土壤。

申请人　广西林业科学研究院

选育人　王以红、吴幼媚、蔡　玲、黄宏喜等

编号：（桂 SY ）第**022**号　　发证机关　广西壮族自治区林木品种审定委员会

2011 年 **12** 月 **31** 日
