# Supplementary material for: Metabolic acclimation supports higher aluminium-induced secretion of citrate and malate in an aluminium-tolerant hybrid clone of Eucalyptus
Source: BMC Plant Biol. 2021 Jan 6;21:14. doi: 10.1186/s12870-020-02788-4 (PMC7789223; doi:10.1186/s12870-020-02788-4)
Supplement: Supplementary file 3 — Additional file 3. Certification of Eucalyptus from Guangxi forestry research institute. [file 12870_2020_2788_MOESM3_ESM.pdf]

Title：Certification of *Eucalyptus* from Guangxi forestry research institute.

Certification

To whom it may concern:

The two clones of *Eucalyptus*，*E. grandis*× *E. urophylla* GL-9 (marked as "G9")and *E. urophylla* GL-4 (marked as "W4") were propagated and bred by Eucalyptus research department of Guangxi forestry research institute. Guodongqiang, a senior engineer and researcher undertook the formal identification of the *Eucalyptus* clone materials used in this manuscript “Metabolic acclimation supports higher aluminium-induced secretion of citrate and malate in an aluminium-tolerant hybrid clone of *Eucalyptus*” that will be submitted to BMC plant biology. *E. grandis*× *E.urophylla*GL-9 and *E. urophylla*GL-4 has been kept in Guangxi forestry research institute.

Deposition number of two clones as below:

E. grandis× *E. urophylla* GL-9 桂 S-SC-EGU-023-2011

E. urophylla GL-4 桂 S-SC-EU-022-2011

Hereby certified.

Guo dongqiang

13/05/2020

Guangxi forestry research institute

广西壮族自治区林业科学研究院

13/05/2020
